# The Impact of a Comprehensive Sexual Health Education Program on STI Knowledge and Attitudes among Philadelphia’s Proud Teens

**DOI:** 10.1007/s10900-026-01591-w

**Published:** 2026-06-18

**Authors:** Priya Nigam, Jade Truehart, Lin Zhu, Yin Tan, Min Qi Wang, Aisha Bhimla, Julia N. Trout, Thoin Begum, Eliza Davison, Grace X. Ma

**Affiliations:** 1 Center for Asian Health, Department of Population Health Sciences, Lewis Katz School of Medicine, Temple University, Philadelphia, PA USA; 2 School of Public Health, University of Maryland, College Park, MD USA; 3 Germantown Friends School, Philadelphia, PA USA

**Keywords:** Sexually transmitted infections, Underserved populations, Sexual health education, Informed consent, Adolescent, Condoms, Unsafe sex

## Abstract

Approximately 25% of sexually transmitted infections (STIs) in Philadelphia involve teens and birth rates among underserved youth is more than double the national average. Proud Teens of Philly (PTOP), a community-based youth-oriented sexual-health education program, aims to mitigate disparities among underserved youth, including Latino/Hispanic and Black youth. This study assessed the impact of PTOP on sexual-health education and behaviors among Philadelphia youth. Participants were recruited from schools, churches, and community-based organizations, to attend eight one-hour-long virtual synchronous sessions. Evidence-based sexual-health curricula, Positive Potential and Making Proud Choices, were administered to 650 Philadelphia students aged 12–18 during the 2021–2022 academic year. Pre- and post-surveys were administered electronically to collect data on demographics, sexual attitudes, HIV/STI knowledge, contraception knowledge and access, confidence in consent and condom use, and life skills. Paired t-tests comparing pre- and post-survey responses were conducted. Participants demonstrated significant improvement in knowledge (t = 10.48, *p* < 0.0001) regarding STI transmission, HIV pre-exposure prophylaxis, and birth control. They felt more prepared to reject unprotected sex (t = 4.81, *p* < 0.0001) and better understood the efficacy of condoms. PTOP curricula increased exposure to alternative forms of contraception. In addition, students gained valuable skills in communication, goal setting, and emotion management. Engagement in a comprehensive sexual-health education program can significantly improve STI knowledge, sexual attitudes, and consent among underserved youth populations. Increased knowledge and emotion management empower teens to make safer choices, potentially limiting engagement in and adverse outcomes of risky sexual behaviors. Future studies should explore longitudinal impacts of youth-oriented sexual-health educational programs.

## Introduction

Philadelphia experiences disproportionately higher incidences of teen pregnancy, human immunodeficiency virus (HIV) infection, and sexually transmitted infections (STIs), such as chlamydia and gonorrhea. The highest burden of HIV in Philadelphia is among non-Hispanic Black individuals, followed by the Latino/Hispanic population [1]. In fact, 69.3% diagnosed with acquired immunodeficiency syndrome in 2020 in Philadelphia were Black. Moreover, Philadelphia has the third highest STI rates among United States metro areas, with approximately 25% of STI cases in Philadelphia occurring among teens aged 15–19 [2, 3]. In 2019, an estimated 49% of teens reported using a condom in their last sexual encounter; this value has declined steadily since 2001 [2].

Because of the extremely high STI burden, local resources in the greater Philadelphia region tend to focus on access to screening and treatment, rather than prevention. Despite improvements in contraception, information accessibility, and cultural awareness, there is also a significant burden of teen pregnancy. Philadelphia’s teen birth rate is 28.8 per 1,000. While overall teen birth rates have declined, Hispanic and Black teens are still disproportionately affected. Sexual-health education is often a small slice of a larger district-wide curriculum, and resources outside of schools and churches typically focus on supporting teen mothers and reducing repeat pregnancies. This reactionary approach ignores risk-avoidance and prevention, resulting in an absence of funding for mitigation efforts. Consequently, the lack of proactive, comprehensive sexual-health education in Philadelphia leaves youth vulnerable to STIs and teen pregnancy. To achieve sustainablo9e change, Philadelphia must implement a systemic approach that focuses specifically on risk reduction.

Previous research indicates that low socioeconomic status is strongly associated with STI and teen pregnancy incidence. In Philadelphia, 1 in 3 children live in poverty and 1 in 2 live in a single-parent household [2]. STI diagnosis is associated with racial disparities, especially among young people [4]. Limited educational opportunities, frequent housing relocation, and unstable employment also affect youth and family health. Chronic strain imposed by such factors can impact the body’s stress response [5], precipitating the development of chronic illnesses and poorer reproductive health outcomes.

Previous sexual-health intervention programs have shown that increased knowledge about sexual-health can empower individuals to make safer choices. After access to a teen pregnancy prevention mobile application, for example, Black and Latina women had better contraceptive knowledge, were less likely to report having sex without contraception, and felt more confident in their ability to use birth control during every sexual encounter [6].

Proud Teens of Philly (PTOP) is a youth-oriented sexual-health education program that aims to mitigate health disparities among underserved youth, especially Latino/Hispanic and Black youth, ages 12–19 in the greater Philadelphia area. PTOP adopts a youth-driven and multi-system approach by combining school- and evidence-based curricula [*Making Proud Choices* (MPC) and *Positive Potential* (PP)] with social media and community outreach. Participation in this program stands to improve knowledge and change attitudes regarding STIs, contraception, and consent. This study aims to explore how sexual knowledge and attitudes changed from pre- to post-PTOP implementation during the second year of the PTOP project, based on the comparison of survey results before and after implementation. Moreover, this study seeks to demonstrate that comprehensive sexual-health education can eventually decrease adverse outcomes of risky sexual behaviors.

## Methods

### Recruitment

Throughout the project period, we used community-based methods to engage youth and their communities to take part on the PTOP program. We identified and reached out to middle and high schools, including public, charter, and language-based schools as well as community-based organizations serving youth in Philadelphia. Program flyers were shared with coordinators at these organizations to share and gauge interest among youth and their parents. Recruitment of youth was done through teacher, community stakeholder, counselor, and staff involvement, and through self-volunteering through school/community-based organization activities. This included events, classes, after school programming, and social media where students were introduced to preventative services available through PTOP. We partnered with coordinators at each site to schedule and carry out the program and provided flexibility in deciding the scheduling, format/structure, and other important details for successful and effective program delivery.

### PTOP Program

PTOP is a sexual-health program intended to equip Philadelphia youth with the tools and attitudes to be healthy adults. Currently, local resources focus on supporting teens who already experience challenging health outcomes, with few resources devoted to educating young people on maintaining sexual health. While STI testing and treatment is available, there is little focus on risk-avoidance or prevention. For long-term, sustainable education efforts, systemic change that focuses on proactive risk-reduction and risk-avoidance strategies is necessary. Successful implementation of PTOP tools is expected to raise the onset age of sexual activity and improve life skill development for practicing healthy choices. These changes can help reduce overall sexual risk-taking behaviors and health disparities among Philadelphia’s underserved teens.

By developing a network of youth-focused agencies to support whole-child development, PTOP aims to reduce teen pregnancy and adverse adolescent health outcomes. These outcomes are grounded in the forces affecting underserved Philadelphia communities, including social determinants of health such as unstable family structures, cultural norms, pervasive poverty, and low-resourced neighborhood and built environments. High school students were administered the MPC curriculum, while middle school students were administered the PP curriculum. The MPC curriculum is an evidence-based, eight-module course that provides knowledge and confidence to reduce the risk of STIs, HIV, and pregnancy using safe sex approaches [7]. It utilizes interactive exercises and small group discussion to cover adolescent goals and dreams, STI transmission and prevention, teenage pregnancy, beliefs and attitude development, and condom-related skills and self-efficacy development [7]. The PP curriculum is a six-week evidence-based program geared toward middle school students [8]. It focuses on reducing risky youth behavior by providing age-appropriate education on bullying, self-confidence, and positive youth development [8]. Both curricula were modified such that PTOP could be delivered virtually due to the COVID-19 pandemic.

We employed multiple strategies to ensure that youth taking part in the program continued to be engaged in the program. Some methods of engagement included using interactive games and activities in sessions, providing branded merch and raffle prize giveaways, being available outside of the curriculum as a form of social support, and encouraging open discussion within each session. The PTOP program also held both community advisory and youth advisory boards in which the team obtained feedback from members about how to continue engaging youth and preventing attrition.

### Study Sample

The surveys were collected from an ongoing PTOP program during both first (*n* = 588) and final (*n* = 322) implementation sessions, using self-administered digital survey forms. Cumulatively, 650 PTOP participants, consisting of middle school and Philadelphia charter high school students between ages 12 and 18, completed surveys. Surveys were administered during the 2021-22 academic school year. Participants completed the surveys anonymously and omitted any questions they did not feel comfortable answering. Due to differences in survey administration at some sites, three sites were excluded from data analysis; students who did not complete both the pre-survey and post-survey were not included; as well, middle school students only received the post-survey, since the PTOP curriculum was often their first exposure to any of these topics and thus were not included in the final sample.

### Survey Administration and Measures

Participants in MPC were electronically administered a pre-program implementation survey to assess baseline knowledge and a post-program survey to assess their growth. PP participants only completed a post-program survey. Data were collected on demographics, sexual attitudes, HIV/STI knowledge, contraception knowledge and access, confidence in consent and condom use, and life skills.

We examined the demographic distribution of the pre- and post-survey populations using means, median, and mode with univariate descriptive statistics. We assessed the completeness of the data and excluded any participants who failed to do either the pre or post survey. The data from three charter school sites (*n* = 202) were also removed from our analysis due to inconsistencies in survey administration. The analysis in this paper was thus limited to high school students (*n* = 426), as middle school students only completed the post-survey. For any reverse-coded questions, we converted the responses to match the one-to-five numerical scale of the other questions, such that a higher score always correlated to improved attitudes or knowledge. A p-value of 0.05 or below was considered statistically significant. We performed paired t-tests on sexual attitudes, HIV/STI knowledge, contraception knowledge and access, confidence in consent and condom use, and life skills. For HIV/STI knowledge and life skills, paired t-tests were performed for each item, as well as for a summed score. Otherwise, each item in the survey was analyzed individually. The statistical analysis was carried out using SASv9.04.

## Results

Of the 650 middle and high school students involved in the second year of PTOP, 426 high school students completed both the pre and post survey and were used for general analysis. Table 1 demonstrates demographic factors of PTOP participants after excluding middle school students, participants with incomplete surveys, and participants at sites with improper survey administration. In total, 169 cis-gender males and 234 cis-gender females were involved in the study, in addition to 14 gender-fluid, 1 transgender male, 1 transgender female, and 7 non-binary identifying participants. The predominant age of participants was fifteen (34%), but participants ranged from 12 to 17 years of age. Approximately 52% of participants were in 9th grade and 38% were in 10th grade, although grades 9 to 12 were represented. Approximately 64% of participants were non-Hispanic Black and 22% of participants identified as Latino/Hispanic.

**Table 1:** Demographic characteristics of Proud Teens of Philly Year 2 cohort (*n* = 426)

Demographic Factor	*N* (%)
Gender Identity	
Cis-Gender Male	169 (39.7)
Cis-Gender Female	234 (54.9)
Transgender Male	1 (0.23)
Transgender Female	1 (0.23)
Gender-fluid	14 (3.29)
Non-Binary	7 (1.64)
**Age (years)**	
12	1 (0.23)
13	32 (7.51)
14	114 (26.8)
15	146 (34.3)
16	123 (28.9)
17	10 (2.35)
**Grade**	
9th Grade	231 (51.6)
10th Grade	171 (38.2)
11th Grade	22 (4.91)
12th Grade	2 (0.45)
**Race/Ethnicity**	
Non-Hispanic Black	272 (63.9)
Non-Hispanic White	9 (2.11)
Non-Hispanic Asian/NHPI/American Indian and Alaska Native/AIAN	11 (2.58)
Non-Hispanic Multi-Race	32 (7.51)
Non-Hispanic Other/Don’t Know	7 (1.64)
Hispanic Black	36 (8.45)
Hispanic White	9 (2.11)
Hispanic Asian/NHPI/AIAN	10 (2.35)
Hispanic multi-race	9 (2.11)
Hispanic Other/Don’t Know	31 (7.28)
**Latino/a/Hispanic**	
Hispanic	95 (22.3)
Non-Hispanic	331 (77.7)

Abbreviations: AIAN, American Indian and Alaska Native; NHPI, Native Hawaiian & Pacific Islander

Table 2 quantifies results of the 13-item sexual knowledge portion of the pre- and post-surveys. Participants showed significant improvement in knowledge on all tested subjects, both summed (t-test score = 10.48, p-value < 0.001) and individually, including STI transmission, HIV misconceptions, pre-exposure prophylaxis for HIV (PrEP), affirmative consent, and birth control. For example, participants were better equipped to dispel incorrect statements such as “If you feel healthy, you don’t have an STI” (t-test score = 6.07, p-value < 0.001). Participants also developed better understanding of HIV transmission, recognizing that “HIV is present in the blood, semen, and body fluid” (t-test score = 5.15, p-value < 0.001). Importantly, participants gained knowledge regarding the transmission of STIs congenitally (t-test score = 6.94, p-value < 0.001) and the immunocompromising effect of HIV (t-test score = 4.66, p-value < 0.001). There was an increase in knowledge on HIV transmission via sex with a person who uses drugs (t-test score = 9.35, p-value < 0.001) and on the lack of HIV transmission through sharing a sink or toilet seat (t-test score = 8.50, p-value < 0.001). Students showed improved understanding of the use of PrEP to reduce their chances of contracting HIV if exposed (t-test score = 6.62, p-value < 0.001). Participants also improved perceptions regarding birth control options, understanding that this included both barrier protection and hormonal options (t-test score = 4.12, p-value < 0.001). There was no significant improvement in knowledge regarding HIV transmission and pregnancy-related stigma, but notably these topics had high baseline knowledge.

**Table 2:** Change in knowledge and attitudes regarding STIs before and after the educational intervention among the Proud Teens of Philly Year 2 cohort (*n* = 426)

T/F STI Statement	Pre-Score	Post-Score	t-test	*p*-value
Burning while urinating can be a common symptom of STIs.	251 (67.5)	151 (71.2)	3.31	*0.001*
If you feel healthy, you don’t have an STI.*	232 (62.5)	180 (84.5)	6.07	*< 0.001*
A pregnant woman who has an STI can give it to her baby.	204 (55.3)	173 (81.2)	6.94	*< 0.001*
Having HIV/AIDs makes you more likely to get other diseases.	162 (43.9)	122 (57.3)	4.66	*< 0.001*
A person can have HIV/AIDs and give it to other people even if the person does *not* look sick.	292 (78.7)	179 (84.4)	1.69	*0.09*
Having sex with a person who uses drugs is a way of getting HIV/AIDs.	81 (21.8)	120 (56.3)	9.35	*< 0.001*
There is a good chance of getting HIV/AIDs from sharing a sink, shower, or toilet seat with someone who has HIV/AIDs.	116 (31.3)	150 (71.1)	8.50	*< 0.001*
HIV is present in the blood, semen, and body fluid.	253 (68.2)	189 (85.7)	5.15	*< 0.001*
A person at-risk for HIV can take one PREP to reduce the chance of contracting HIV.	146 (39.5)	134 (63.5)	6.62	*< 0.001*
People must agree to the sexual behaviors they engage in (“yes means yes”).	269 (72.3)	176 (83.0)	3.10	*0.002*
If a partner says “I don’t know,” or “maybe” that is affirmative consent.*	225 (61.1)	162 (76.4)	3.49	*0.001*
Birth control can be hormonal or barrier protection.	201 (54.0)	142 (67.3)	4.12	*< 0.001*
If a girl and boy have sex, the girl should be more responsible for preventing pregnancy than the boy. *	232 (62.9)	154 (72.6)	0.88	*0.38*
**Summary Score (N%)**	2664 (55.3)	2032 (74.0)	10.5	*< 0.001*

Abbreviations: STIs – sexually transmitted infections; HIV – human immunodeficiency virus; AIDs – acquired immunodeficiency syndrome; * items are reverse scored

Table 3 depicts the improvements in confidence in sexual communication after PTOP participation. Involvement in PTOP significantly improved confidence in saying no to sex if a partner would not use a condom (t-test score = 3.20, p-value = 0.001). Participants already felt comfortable talking to partners about condom use (t-test score = 1.27, p-value = 0.21), verbally consenting to sex (t-test score = 1.47, p-value = 0.14), and asking for consent for sex (t-test score = 1.50,p-value = 0.13) prior to program participati.

**Table 3:** Changes in Perceived Ability to Consent, Discuss Condom Use, and Sexual Attitudes

Confidence in Sexual Communication	Pre-Score					Post-Score						
	Strongly Disagree *N* (%)	Disagree *N* (%)	Neutral/ I don’t know *N* (%)	Agree *N* (%)	Strongly Agree *N* (%)	Strongly Disagree *N* (%)	Disagree *N* (%)	Neutral/ I don’t know *N* (%)	Agree *N* (%)	Strongly Agree *N* (%)	t-score	*p*-value
Talking to a partner about using condoms	14 (3.76)	18 (4.84)	60 (16.1)	139 (37.4)	141 (37.9)	8 (3.8)	6 (2.83)	29 (13.7)	72 (34.0)	97 (45.8)	1.27	*0.21*
Saying no to sex if a partner won’t use a condom	27 (7.28)	18 (4.85)	69 (18.6)	78 (21.0)	179 (48.3)	8 (3.78)	3 (1.42)	25 (11.9)	55 (26.1)	120 (56.9)	3.20	*0.001*
Verbally stating consent to someone I want to have sex with	13 (3.46)	14 (3.72)	78 (20.7)	130 (34.6)	141 (37.5)	6 (2.82)	4 (1.88)	36 (16.9)	71 (33.3)	96 (45.1)	1.47	*0.14*
Asking for consent from someone I want to have sex with	11 (2.93)	15 (3.99)	66 (17.6)	144 (38.3)	140 (37.2)	4 (1.88)	5 (2.35)	32 (15.0)	74 (34.7)	98 (46.0)	1.50	*0.13*

Table 4 describes changes in sexual attitudes and preparedness to encourage condom use after PTOP. Students had significantly increased understanding of the efficacy of condoms in preventing pregnancies (t-test score = 3.56, p-value < 0.001). In addition, participants also had significant improvements in understanding the benefits of condoms in preventing STI (t-test score = 2.81, p-value < 0.001) and HIV (t-test score = 4.78, p-value < 0.001) occurrence and transmission. Students felt significantly more prepared to respond in situations where their partner may give excuses for not wanting to use a condom (t-test score = 4.81, p-value < 0.001).

**Table 4:** Changes in Sexual Attitudes and Preparedness to Encourage Condom Use

Condom Benefits	Pre-Score					Post Score								
	Strongly Disagree *N* (%)	Disagree *N* (%)	Neutral/ I don’t know *N* (%)	Agree *N* (%)	Strongly Agree *N* (%)		Strongly Disagree *N* (%)	Disagree *N* (%)	Neutral/ I don’t know *N* (%)	Agree *N* (%)	Strongly Agree *N* (%)	t-score		*p*-value
Prevent pregnancy	29 (6.84)	36 (8.49)	62 (14.6)	200 (47.2)	97 (22.9**)**		7 (3.29)	7 (3.29)	11 (5.16)	100 (47.0)	88 (41.3)	3.56		*< 0.001*
Prevent STIs (HPV, Gonorrhea, Chlamydia, Herpes)	27 (6.40)	30 (7.11)	86 (20.4)	155 (36.7)	124 (29.4)		6 (2.83)	10 (4.72)	13 (6.13)	99 (46.7)	84 (39.6)	2.81		*< 0.001*
Prevent HIV transmission	45 (10.7)	31 (7.40)	92 (22.0)	150 (35.8)	101 (24.1)		6 (2.84)	7 (3.32)	14 (6.64)	96 (45.5)	88 (41.7)	4.78		*< 0.001*
Confidence in addressing excuses against condom use	48 (11.6)	30 (7.25)	133 (32.1)	116 (28.0)	87 (21.0)		8 (3.77)	5 (2.36)	34 (16.0)	82 (38.7)	83 (39.2)	4.81		*< 0.001*

Abbreviations: HIV, human immunodeficiency virus; HPV, human papilloma virus; STIs, sexually transmitted infection

**Fig. 1:**
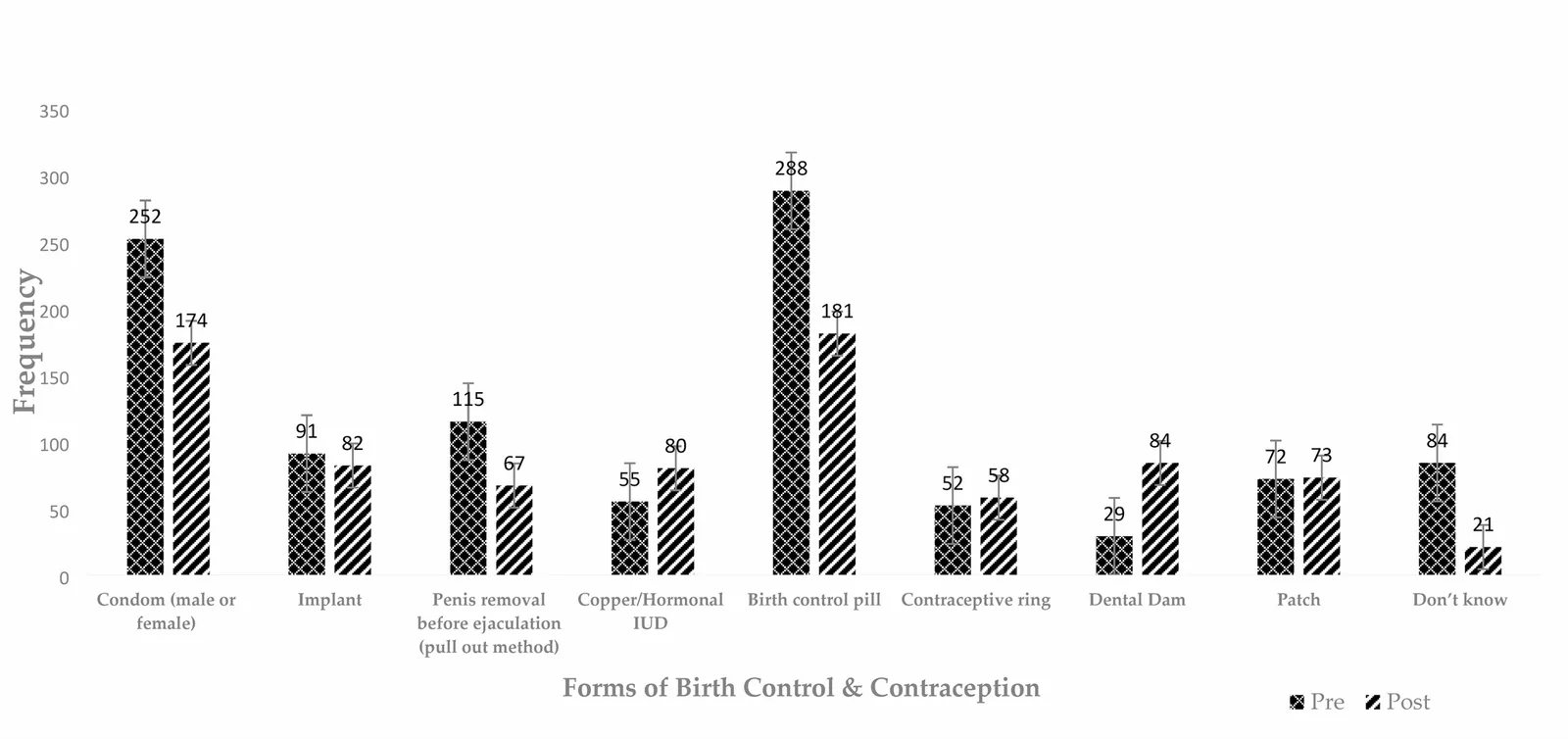
Changes in Identification of Contraception and Birth Control

Figure 1 demonstrates improvements in birth control and contraception knowledge and familiarity. Participants had high baseline knowledge of the birth control pill and condoms as pregnancy prevention options. There was a 42% decrease in participants considering penis removal as a contraceptive option after participation, from 115 participants to 67 participants. Knowledge of the dental dam nearly tripled from 29 participants to 84 participants. The number of participants who reported no knowledge of contraceptive methods decreased by 75%, from 84 to 21 participants. PTOP also improved knowledge of birth control access. Only 36.49% of participants “definitely knew” where to access birth control methods prior to PTOP. In fact, 17.03% reported they did not know where to go for birth control at all. Participants self-reported that their major sources of information about sexual-health and relationships came from school classes, family members, doctors, friends, and the internet. Notably, participants relied the most on family members and relied on doctors and the internet equally.

## Discussion

### Principal Findings

To address the critical gap in comprehensive sexual-health education among underserved youth in Philadelphia, this research study determined the impact of PTOP’s comprehensive sexual-health education on STI knowledge, sexual attitudes, and consent among underserved youth populations in Philadelphia. We found that participants had significant improvements in knowledge regarding transmission of STIs, use indications for PrEP for HIV, and purposes of birth control. They felt more confident rejecting unprotected sex and better understood the efficacy of condoms in pregnancy and STI prevention. These findings underscored the pivotal role of targeted sexual-health education programs in not only enhancing knowledge but also empowering disenfranchised youth to make informed decisions regarding their sexual-health, ultimately contributing to the reduction of health disparities among this vulnerable population in Philadelphia.

Making safer choices requires an understanding of contraceptive options and having confidence in discussing consent and boundaries [9]. After PTOP completion, participants had significant improvement in knowledge of condom benefits. The participants understood the importance of condoms in pregnancy prevention, STI reduction, and HIV transmission prevention. Students felt more comfortable responding to partners who expressed a reluctance to use condoms. More importantly, participants were confident that they could now say no to sex if their partner refused to use a condom. Among sexually active youth across the United States, condom use at their last sexual encounter was only 54.3% [10]. While condoms were the preferred form of contraception in this age group (43.9% of pregnancy prevention methods used), American youth still did not understand the gravity of using condoms to reduce risky outcomes [10]. Condom use decision-making has been found to be heavily influenced by adolescent fears of pregnancy and disease [11]. PTOP has been able to empower teens to use condoms by providing much-needed education about STIs and HIV. After PTOP completion, participants understood how STIs and HIV are transmitted, treated, and prevented.

Many of the participants were familiar with condoms and birth control pills as forms of contraception prior to program involvement, but there was an improvement in knowledge of other forms of contraception, such as intrauterine devices (IUDs), contraceptive rings, and dental dams. Furthermore, participants realized that birth control involved both barrier protection and hormonal contraception. After program participation, the number of youth with no contraceptive knowledge decreased by 75%. Across the United States, birth control pills (23.3%) and alternative birth control methods like IUDs (4.8%) were infrequently used by teens, in comparison to condoms (43.9%), and tended to be used among youth in higher grades [10]. Only 9.1% of teens used both a condom and an IUD, implant, shot, patch, ring, or pill in their last sexual encounter [10]. This may be due to a lack of education, but also a lack of access to quality reproductive care. Black and Latino/Hispanic teens have cited the importance of social-media education, desire for a teen-only clinic, and lack of guaranteed privacy as reasons for underutilization of birth control [12]. Fears regarding birth control side effects and misconceptions about efficacy contributed significantly to underutilization as well [12]. PTOP broke down these barriers by providing open discourse on the effectiveness of each birth control method. Furthermore, the program connects teens to local resources and fosters a social-media community where accurate educational knowledge can be shared. This initiative as part of the PTOP program has been published elsewhere [13].

PTOP provided underserved teen youth in Philadelphia with much-needed exposure to HIV transmission prevention tactics. Students had a significant improvement in PrEP knowledge and learned that they could take one dose of PrEP to reduce their chances of contracting the disease. Unfortunately, 21% of new HIV infections are among 15- to 24-year olds, and more than 40% of youth with HIV are undiagnosed [14]. While more pediatricians need to be asking about sexual practices and HIV exposure, teens also need to be aware of this burgeoning issue. In a U.S. cohort study following youth men-who-have-sex-with-men, one-third of those trying PrEP discontinued it and 79% discontinued without consulting a doctor [15]. More than 18% of these teens cited “not feeling that they are at risk for HIV” as their reason for discontinuation [15]. In addition to eliminating barriers to PrEP access, providers, parents, and teens need to create spaces where this issue can be discussed comfortably. PTOP created a safe environment to discuss PrEP and its importance. Furthermore, PTOP emphasized HIV risk factors and advocated for all sexually active teens to consider using PrEP and obtain regular STI testing.

With PTOP, students gained valuable skills in communication, goal setting, and emotion management, which will aid them in maintaining healthier and positive relationships [7, 16]. PTOP focused on principles of affirmative consent and boundary setting in relationships. Participants felt more confident saying no to engaging in condomless sex after PTOP participation. 11% of young adults (ages 18–24) sexually active before age 20 report not wanting sexual activity during their first sexual encounter [17]. In addition, adolescent girls have historically conveyed sexual refusal through non-verbal cues while adolescent boys proceeded with sexual activity until they heard “no” [18]. This discrepancy has fueled miscommunication during sexual encounters and has led to more adverse events. Furthermore, gender roles and power dynamics have the ability to influence sexual initiation and behavior, including obtaining consent and has been cited to differ among high school male and female students [19]. PTOP taught participants about active and affirmative consent, creating expectations of enthusiastic verbal consent during sexual encounters. By providing the same education to all students, regardless of their gender, any individual involved in a sexual encounter of any type will be empowered to utilize principles of communication and consent.

Notably, the youth in this study already had a high baseline knowledge regarding pregnancy-related stigma and HIV transmission. This may reflect the increased prevalence of popular culture discussing these topics, as well as increased individual exposure due to the rates of single-parent households and HIV in Philadelphia [2, 20]. At baseline, participants already felt comfortable discussing condom use with a partner and asking for consent. This suggests that future curricula can focus on more nuanced sexual-health topics, such as discussing regular STI testing, seeking affirmative consent throughout sexual encounters, and asserting firm boundaries [21].

### Comparison with Existing Literature

Previous studies have explored impacts of programs focused on emotional well-being or sexual-health, but few studies have examined improvements in attitudes and knowledge among individuals in high-risk populations after participation in a comprehensive sexual-health education program. Comprehensive sexual-health education is associated with a lower likelihood of engaging in vaginal intercourse compared to abstinence-only education and with significantly less teen pregnancy compared to no formal sexual education [22]. Some 80% of school-based programs only focused on pregnancy and disease prevention, with no attention on improved social/emotional learning or development of healthy relationships [23]. By focusing on changing sexual attitudes, in addition to improving STI knowledge, the present study equipped youth with tools to make less-risky decisions in the future. Moreover, this study targeted Black and Latino/Hispanic youth, who were under-represented in previous studies but are often most at risk [24].

Due to the internet, teens are more informed today about sexual-health than ever [25]. However, the internet lacks credible sources and often focuses on peer-to-peer information sharing [26]. The existence of comprehensive sexual-health education is vital to ensure that youth are receiving accurate and intentional information that is catered to their situation [25]. PTOP created spaces for youth to understand themselves and their emotional needs. Despite the variety of baseline knowledge of our participants, they all benefited from having a designated opportunity to ask questions and be empowered to make smarter choices.

Teen pregnancy and STI transmission is a widespread issue in Philadelphia and other urban settings [2]. Communities of color continue to face the brunt of these issues due to a lack of infrastructure, a tendency toward reactionary solutions, and issues affecting social determinants of health [27]. System-based programs like PTOP are necessary to proactively prevent adverse outcomes from sexual risk behaviors. Through educational curricula, outreach events, and a strong social-media presence, participants can develop and maintain a strong foundation of knowledge. This study indicates that high-risk teens benefit significantly from early access to comprehensive sexual-health education. Teens learn about sensitive issues in a safe setting, while also developing skills to better cope when sexual situations arise. PTOP teens will be better able to establish consent, obtain contraception if necessary, and assert their boundaries. Thus, PTOP participation will decrease risky sexual behaviors among at-risk youth and decrease the prevalence of STIs and teen pregnancy in this population.

### Strengths and Limitations

While this study has several strengths, it is not without limitations. One limitation involved issues in survey administration at some sites, resulting in students completing surveys incorrectly or incompletely. To minimize the effects of this issue, we removed three sites from data analysis. Another limitation of the study was the reduced sample size. Students who missed the administration of pre-survey or post-survey were not included in the study, even if they attended other PTOP sessions. Middle school students only received the post-survey, since the PTOP curriculum was often their first exposure to any of these topics. As a result, they are not included in the analysis. Additionally, students could opt-out of answering questions that made them feel uncomfortable, which explains inconsistencies in sample sizes between questions. Allowing students to opt-out of answering survey questions also created a safe space for participants, which we feel is immeasurably valuable.

Despite its limitations, we have managed to implement a comprehensive sexual-health curriculum that targets the acute and long-term needs of an underserved population. We provided standardized learning to various sites, even between facilitators, because of our evidence-based and vetted sexual education curriculum. We successfully improved knowledge on STIs, condom use, and pregnancy prevention and have demonstrated the effectiveness of a comprehensive sexual-health education curriculum. Furthermore, many different students and communities were impacted, due to the three-year study length. Therefore, our cumulative results are reliable and valid.

### Future Directions

Given the lack of long-term follow-up, future studies should focus on assessing longitudinal impacts of comprehensive sexual-health education programs and on identifying knowledge gaps for curriculum improvement. PTOP could administer a survey several years after curriculum completion to determine STI prevalence, pregnancy status, and sexual attitudes in this group. Furthermore, it would be useful to conduct focus groups with PTOP participants in the future to identify educational gaps and develop more targeted lesson planning. PTOP could benefit from expanding its community partners so that students can become pro-actively connected with local STI clinics, health centers, and Planned Parenthood.

### Conclusion

Engagement in a comprehensive sexual-health education program such as PTOP can significantly improve STI knowledge, sexual attitudes, and consent among disenfranchised youth. In turn, this will limit the adverse effects of sexual risk behaviors and can empower teens to make safer choices. This study demonstrates the necessity of comprehensive sexual-health education in creating well-informed and well-adjusted teens who will be capable of securing a better future for themselves.

## Abbreviations

HIV: human immunodeficiency virus
AIDs: acquired immunodeficiency syndrome
STI: sexually transmitted infection
PTOP: Proud Teens of Philly
MPC: Making Proud Choices
PP: Positive Potential
PrEP: pre-exposure prophylaxis
IUDs: intrauterine devices
